# Fine Mapping of the Major Histocompatibility Complex Region and Association of the HLA-B*52:01 Allele With Cervical Cancer in Japanese Women

**DOI:** 10.1001/jamanetworkopen.2020.23248

**Published:** 2020-10-29

**Authors:** Tatsuo Masuda, Hidemi Ito, Jun Hirata, Saori Sakaue, Yutaka Ueda, Tadashi Kimura, Fumihiko Takeuchi, Yoshinori Murakami, Koichi Matsuda, Keitaro Matsuo, Yukinori Okada

**Affiliations:** 1 Department of Statistical Genetics, Osaka University Graduate School of Medicine, Osaka, Japan; 2 Department of Obstetrics and Gynecology, Osaka University Graduate School of Medicine, Osaka, Japan; 3 Division of Cancer Information and Control, Aichi Cancer Center Research Institute, Aichi, Japan; 4 Department of Descriptive Cancer Epidemiology, Nagoya University Graduate School of Medicine, Aichi, Japan; 5 Pharmaceutical Discovery Research Laboratories, Teijin Pharma Limited, Hino, Japan; 6 Department of Allergy and Rheumatology, The University of Tokyo Graduate School of Medicine, Tokyo, Japan; 7 Department of Gene Diagnostics and Therapeutics, National Center for Global Health and Medicine Research Institute, Tokyo, Japan; 8 Institute of Medical Science, Division of Molecular Pathology, The University of Tokyo, Tokyo, Japan; 9 Department of Computational Biology and Medical Sciences, The University of Tokyo Graduate School of Frontier Sciences, Tokyo, Japan; 10 Division of Cancer Epidemiology and Prevention, Aichi Cancer Center Research Institute, Aichi, Japan; 11 Department of Cancer Epidemiology, Nagoya University Graduate School of Medicine, Aichi, Japan; 12 Laboratory of Statistical Immunology, World Premier International Research Center Initiative, Osaka University Immunology Frontier Research Center, Osaka University, Osaka, Japan; 13 Institute for Open and Transdisciplinary Research Initiatives, Integrated Frontier Research for Medical Science Division, Osaka University, Osaka, Japan; 14 Now with StemRIM Institute of Regeneration-Inducing Medicine, Osaka University, Osaka, Japan

## Abstract

**Importance:**

Understanding the genetic contribution of the major histocompatibility complex (MHC) region to the risk of cervical cancer (CC) will help understand how immune responses to infection with human papillomaviruses are associated with CC.

**Objective:**

To determine whether the HLA-B*52:01 allele is associated with CC in Japanese women.

**Design, Setting, and Participants:**

This was a multicenter genetic association study. Genotype and phenotype data were obtained from BioBank Japan Project. Additional patients with CC were enrolled from the Aichi Cancer Center Research Institute. An MHC fine-mapping study was conducted on CC risk in the Japanese population by applying a human leukocyte antigen (HLA) imputation method to the large-scale CC genome-wide association study data of using the Japanese population-specific HLA reference panel. Participants included 540 women in BioBank Japan Project with CC or 39 829 women without gynecologic diseases, malignant neoplasms, and MHC-related diseases as controls. An additional 168 patients with CC were recruited from Aichi Cancer Center Research Institute. Histopathological subtypes and clinical stages were not considered. Participants with low genotype call rate, closely related participants, and outliers in the principal component analysis were excluded. Data analysis was performed from August 2018 to January 2020.

**Main Outcomes and Measures:**

Loci within the MHC region associated with CC risk, and the direction and size of association.

**Results:**

A total of 704 CC cases and 39 556 controls were analyzed. All participants were Japanese women with a median (range) age of 67 (18 to 100) years. One of the class I HLA alleles of HLA-B*52:01 was significantly associated with CC risk (odds ratio, 1.60; 95% CI, 1.38-1.86; *P* = 7.4 × 10^−10^). Allele frequency spectra of HLA-B*52:01 are heterogeneous among worldwide populations with high frequency in Japanese populations (0.109 in controls), suggesting its population–specific risk associated with CC. The conditional analysis suggested that HLA-B*52:01 could explain most of the MHC risk associated with CC because no other HLA alleles remained significant after conditioning on the HLA-B*52:01. The HLA amino acid residue–based analysis suggested that HLA-B p.Tyr171His located in the peptide-binding groove was associated with the most significant CC risk (odds ratio, 1.47; 95% CI, 1.30-1.66; *P* = 1.2 × 10^−9^).

**Conclusions and Relevance:**

The results of this study contribute to understanding of the genetic background of CC. The results suggest that immune responses mediated by class I HLA molecules are associated with susceptibility to CC.

## Key Points

**Question:**

Which gene in the major histocompatibility complex region is associated with cervical cancer?

**Findings:**

This genetic association study of 704 women with cervical cancer and 39 829 women without gynecologic disease used fine mapping of the major histocompatibility complex region by human leukocyte antigen imputation and found that HLA-B*52:01 was associated with cervical cancer in the Japanese population.

**Meaning:**

These findings suggest that immune responses against human papillomaviruses are mediated by class I human leukocyte antigen molecules, which are associated with susceptibility to cervical cancer.

## Teaser

This genetic association study uses fine mapping of the major histocompatibility complex (MHC) region by human leukocyte antigen imputation to determine whether the HLA-B*52:01 allele is associated with cervical cancer (CC) in Japanese women.

## Introduction

Cervical cancer (CC) is the fourth most frequently diagnosed cancer and the fourth-leading cause of cancer death in women worldwide in 2018.^1^ Although environmental factors such as smoking, parity, and oral contraceptive use are known to be associated with CC,^2^ it is well established that persistent infection with high-risk human papillomaviruses (HPVs) has a major carcinogenic role.^3^ Generally, although exposure to HPVs is highly prevalent, most women infected with HPVs do not acquire persistent infection.^4,5^ Because persistent infection is supposed to be mediated by individuals’ immune responses, CC can be considered as an immune-related disease. The major histocompatibility complex (MHC) region, which regulates immune responses to pathogens, is expected to have an essential role in controlling HPV infection.

Recent genome-wide association studies (GWASs)^6,7,8,9^ have suggested that genetic variants within the MHC region are associated with CC. However, because the genetic structure of the MHC region is highly complex and diverse among populations,^10,11,12^ more detailed and population-specific analysis is required for fine mapping of the CC causal variant embedded within MHC. Because the previous candidate gene-based reports have mainly focused on the class II human leukocyte antigen (HLA) alleles,^13,14,15,16,17,18,19,20^ comprehensive fine-mapping analyses assessing both class I and II HLA alleles, nonclassical HLA alleles, and amino acid variations are warranted.^21^ Recently, a new computational method has been developed for detailed fine-mapping analysis of the MHC region, which is called the HLA imputation.^10,11,12,21^ By using the population-specific reference panel, application of HLA imputation can achieve fine mapping of the causal HLA variants of the human complex traits.^10,11,12,21^

This study aimed to elucidate the CC-related genetic risks associated with the MHC region in the Japanese population. By applying HLA imputation to the large-scale CC GWAS data with the latest population-specific HLA reference panel of 1120 individuals from the Japanese population,^10,11,12^ our study used fine mapping of an HLA allele associated with CC risk. To our knowledge, this study is the first region-wide comprehensive association study among the Japanese population and can be a replication of the previous European study.^21^

## Methods

### Participants

Recruitment started from 2003 and ended in 2013. The data were collected at the timing of recruitment, and the participants were followed up until 2018. We enrolled 708 women with CC, including women with cervical intraepithelial neoplasias, and 39 829 women in the control group. Of these, 540 cases and 39 829 controls were from Biobank Japan Project (BBJ),^7^ and 168 cases were from the Aichi Cancer Center Research Institute (separated into 2 data sets, Aichi1 [96 cases] and Aichi2 [72 cases]. CC was diagnosed according to histopathological findings, whereas histopathological subtypes, including the extent of the disease, were not considered. To avoid potential confounding, we excluded control women who had other malignant neoplasms or diseases thought to be strongly associated with the MHC region.^7^ This study was approved by the ethical committee of Osaka University Graduate School of Medicine. All of the participants from BBJ provided written informed consent as approved by the ethical committee of RIKEN Yokohama Institute and the Institute of Medical Science, the University of Tokyo. All of the participants from Aichi provided written informed consent as approved by the ethical committee of Aichi Cancer Center Research Institute. This study followed the Strengthening the Reporting of Genetic Association Studies (STREGA) reporting guideline.

### Genotyping and Quality Control

The genomic DNA was prepared in accordance with the standard protocols provided by the manufacturer. The GWAS data sets were genotyped by using either of the following single-nucleotide variant (SNV) (formerly SNP) microarrays: the Illumina HumanOmniExpressExome BeadChip or a combination of the Illumina HumanOmniExpress and HumanExome BeadChips (for the BBJ participants), or Illumina HumanOmniExpress BeadChip (for both data sets of Aichi1 and Aichi2). Because of the imbalance of sample size and different SNV arrays used for genotyping among different data sets, we applied the stringent quality control (QC) filters separately to each data set as previously described.^7^ Briefly, we first applied the QC filters of the participants. We excluded the samples with low call rates (<0.99 for BBJ, <0.95 for Aichi1, and <0.97 for Aichi2). We then applied the QC filters of the variants: (1) exclusion of the variants with low call rates (<0.99 for BBJ and <0.97 for Aichi1 and Aichi2), (2) exclusion of the variants with allele frequency discordances among data sets (>0.20 in any data set pairs), and (3) exclusion of the variants within case association with *P* < 1.0 × 10^−7^. We also excluded indels and the variants with duplicated positions. We then combined the GWAS genotype data and applied further QC filters. To avoid bias due to relatedness among participants and population stratification, we applied QC filters to the participants as follows: (1) exclusion of the closely related samples (identity by descent PI_HAT calculated to be greater than 0.125 by PLINK version 1.90b3.3 [Center for Human Genetic Research, Massachusetts General Hospital, and the Broad Institute of Harvard and MIT]), and (2) exclusion of outliers in principal component analysis conducted with the multiethnic 2504 samples from the 1000 Genome Project phase 3v5a. Finally, we applied the QC filters of the variants: (1) exclusion of the variants with low minor allele frequencies (MAF) (<0.01 in either of the cases or controls), and (2) exclusion of the variants with *P* values for departure from Hardy-Weinberg equilibrium (HWE) <1.0 × 10^−6^. The genotypes were phased using SHAPEIT2 software version 2.r837 (Conservatoire National des Arts et Métiers [CNAM] and University of Oxford). Principal component analysis (PCA) was performed using EIGENSOFT software version 6.1.4 (David Reich Lab) with linkage disequilibrium–pruned genome-wide SNV genotypes. After exclusion of the QC-filtered subjects, we recalculated the principal components in the same manner, which were then used as the covariates in the association analysis. Data manipulation was performed using PLINK software version 1.90b3.3 (Center for Human Genetic Research, Massachusetts General Hospital, and the Broad Institute of Harvard and MIT).

### HLA Imputation

We adopted the HLA reference panel of the Japanese population (1120 individuals) constructed in the previous studies.^10^ We converted the original reference data into a vcf format. We extracted the variants located in the MHC region (defined here as from 24 to 36 Mbp on chromosome 6, Genome Reference Consortium Human Build 37 [GRCh37]) from the GWAS data, and applied HLA imputation using Minimac3 software version 1.0.11 (Abecasis Lab) and the reference panel. Variants with MAF greater than or equal to 0.01 in both case and control subjects, and imputation quality information estimated *r*^2^ greater than or equal to 0.5 were selected for the following analyses.

### Statistical Analysis

As described previously,^10^ associations of the HLA variants as exposures with CC as an outcome were evaluated using logistic regression model implemented in R statistical software version 3.5.1 (R Project for Statistical Computing). We assumed additive effects of the allele dosages on the log-odds scale, and age, square age, body mass index (calculated as weight in kilograms divided by height in meters squared), and top 20 principal components were used as covariates to avoid possible confounding. We defined the HLA variants as biallelic SNVs in the MHC region, 2-digits and 4-digits biallelic HLA alleles, biallelic HLA amino acid variants corresponding to their respective residues, and multiallelic HLA amino acid variants for each amino acid position.

For multiallelic amino acid variants, we estimated its significance by an omnibus test for each amino acid position by a log-likelihood ratio test, comparing the likelihood of the fitted model with the null model. The significance in improvement of the model fitting was evaluated by the deviance, which follows χ^2^ distribution with *m *− 1 *df* for an amino acid position with *m* polymorphic residues. The genome-wide significance threshold (*P* < 5.0 × 10^−8^) was adopted to control the risk of false positive findings (α = .05), and testing was 2-sided.^22^ The conditional association analysis of the HLA variants was conducted by additionally including the lead HLA variant genotype dosage as a covariate. Data analysis was performed from August 2018 to January 2020.

## Results

### Participants

After applying the stringent QC filters excluding samples with low call rate, closely related subjects, and outliers in PCA, we obtained a total of 704 participants with CC and 39 556 participants in the control group. All participants were Japanese women with a median (range) age of 67 (18 to 100) years (Table). The CC cases consisted of 538 cases from BBJ and 166 cases from the 2 Aichi cohorts. The women in the control group did not have any malignant neoplasms or known MHC-related diseases.

**Table.** Participant Characteristics

Characteristics	Participants			
	BBJ		Aichi	
	With CC	No CC	Subset 1	Subset 2
Recruited, No.	540	39 829	96	72
Eligible, No.	538	39 556	94	72
Age, median (range), y	54 (24-92)	67 (18-100)	41 (27-77)	44 (26-75)
Body mass index, median (range)^a^	21.6 (15.2-35.8)	23.0 (11.4-55.2)	21.2 (18.0-30.8)	20.4 (16.6-28.9)

Abbreviations: BBJ, Biobank Japan; CC, cervical cancer.

^a^ Body mass index is calculated as weight in kilograms divided by height in meters squared.

### Genotyping, Quality Control, and HLA Imputation

After applying the QC filters of variants, excluding variants with low call rate, discordant allele frequency among cohorts, duplicated positions, low MAF (<0.01), deviating from HWE, and indels, we obtained 460 666 variants. Of these, 4049 variants located in the MHC region (defined from 24 to 36 Mbp on chromosome 6, NCBI build 37) were extracted for HLA imputation. By performing HLA imputation based on the large-scale population-specific HLA reference panel of Japanese individuals, we obtained imputed genotype of the 141 2-digits HLA alleles, 199 4-digits alleles, 1631 amino acid residues at 1385 polymorphic positions, and 3276 SNVs in the MHC region.

### Association Analysis and Conditioning

**Figure 1.**
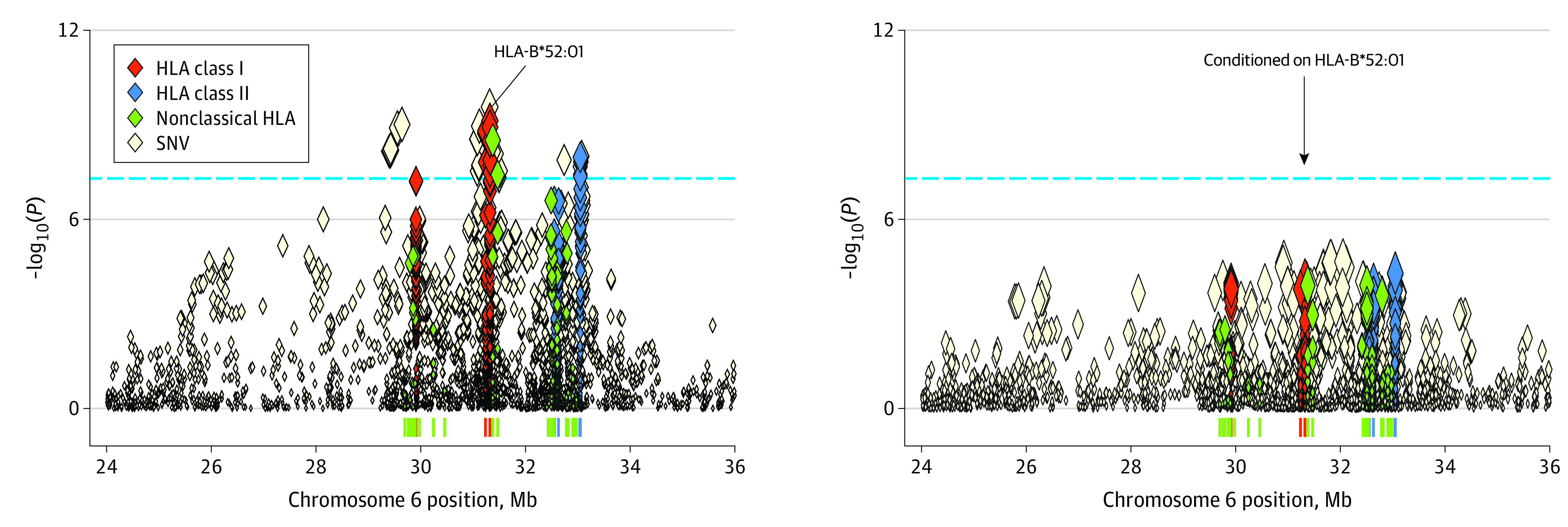
Regional Associations of the Variants in the Major Histocompatibility Complex Region With Cervical Cancer Risk Regional associations of the variants in the major histocompatibility complex region with cervical cancer risk in the Japanese population estimated on the basis of the human leukocyte antigen (HLA) imputation analysis. Left, Nominal regional associations. Right, Regional associations conditioned on HLA-B*52:01. Each diamond represents the −log_10_(*P*) of the variants, including single-nucleotide variant (SNV); 2-digit, 4-digit, and 6-digit HLA alleles; and amino acid variants of HLA genes. The dashed horizontal line represents genome-wide significance threshold of *P* = 5.0 × 10^−8^.

**Figure 2.**
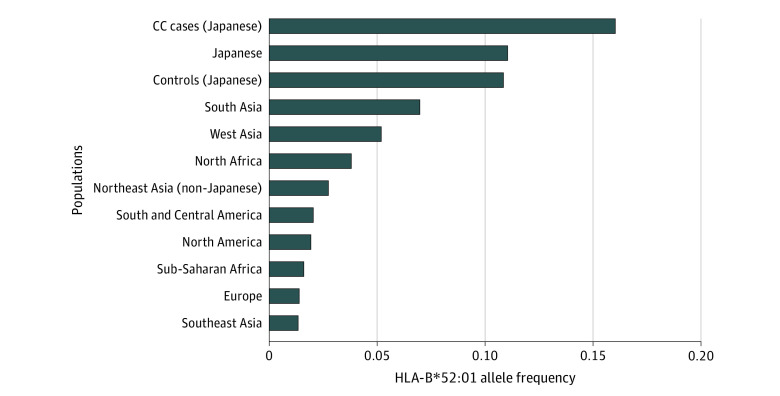
HLA-B*51:01 Allele Frequency Spectra in Worldwide Populations Mean HLA-B*52:01 allele frequencies among each population is plotted in the bar-plot. HLA allele frequency data were obtained from Allele Frequency Net Database (eAppendix in the Supplement). Cervical cancer (CC) cases (Japanese) represents the CC case population enrolled in the current study.

We conducted logistic regression analysis of the HLA alleles and SNVs within the MHC region assuming additive effects of the allele dosages on the log-odds scale. We also conducted an omnibus test estimating the significance of amino acid variants for each amino acid position by a log-likelihood ratio test. The regional association results are shown in Figure 1. The most significant association was observed on the SNV located in the MHC class I region (rs2844586, odds ratio [OR] = 1.58; 95% CI, 1.37-1.83; *P* = 2.7 × 10^−10^; imputation quality information *r^2^* = 0.98), which was in strong linkage disequilibrium with HLA-B*52:01 (*r^2^* = 0.90 in Japanese). Among the HLA alleles, the most significant association was observed at HLA-B*52:01. The frequency of the HLA-B*52:01 variant was 0.160 in the CC group and 0.109 in the control group (OR = 1.60; 95% CI, 1.38-1.86; *P* = 7.4 × 10^−10^) (Figure 1A). HLA-B*52:01 is a common HLA-B allele in the Japanese population with a frequency of 0.110 (Figure 2), and is included in a Japanese population-specific common long-range haplotype.^11^ On the other hand, the HLA alleles previously reported to have associations with CC did not reach the genome-wide significance. Associations of HLA-B*15, HLA-B*15:01, HLA-DRB1*11, HLA-DRB1*11:01, HLA-DRB1*13, HLA-DRB1*13:02, HLA-DRB1*15, HLA-DQB1*06:01, and HLA-DQB1*06:04 were not significant but were replicated with directional concordance of the allelic CC risk (eTable 1 in the Supplement).^13,14,16,17,18,19,20,21^

When conditioned on HLA-B*52:01, no other variants in the MHC region satisfied the genome-wide significance (Figure 1B). This suggested that all the other variants that originally satisfied the genome-wide significance were in linkage disequilibrium with HLA-B*52:01. Any of the previously reported CC-risk HLA variants did not indicate genome-wide significance after conditioning. These observations suggested that HLA-B*52:01 would be the most probable causal allele that could explain the majority of the CC risk associated within the MHC region in Japanese women. Of the aforementioned replicated HLA alleles, HLA-B*15:01, HLA-DRB1*11, HLA-DRB1*11:01, HLA-DRB1*13, HLA-DRB1*13:02, HLA-DRB1*15, and HLA-DQB1*06:04 failed to maintain their association after conditioning on HLA-B*52:01, suggesting that these associations might have been driven by HLA-B*52:01. Further study is required to elucidate the association of these HLA alleles.

**Figure 3.**
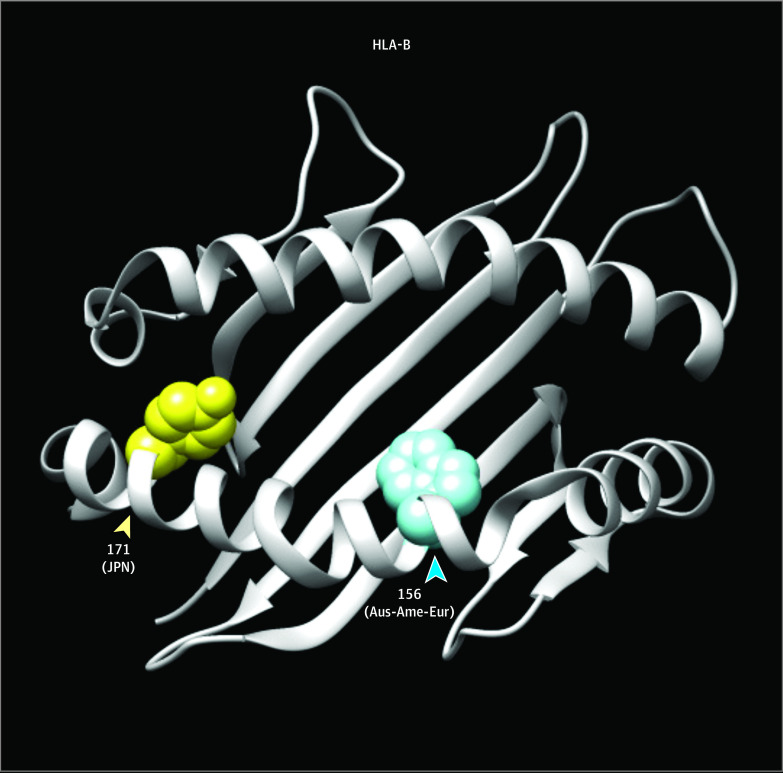
Cervical Cancer (CC) Risk Amino Acid Positions of HLA-B Molecule in 3-Dimensional Ribbon Model HLA amino acid positions associated with CC risk in HLA-B molecule are indicated in a 3-dimensional ribbon model. The protein structure of HLA-B is based on Protein Data Bank entries 1XR8, and prepared using UCSF Chimera version 1.11.2 (Resource for Biocomputing, Visualization, and Informatics). Residues at amino acid positions associated with CC risk in Japanese (JPN this study) and Australian-American-European (Aus-Ame-Eur)^21^ are highlighted in yellow and blue, respectively.

At an amino acid level, the most significant association was observed at the amino acid position 171 of the HLA-B molecule (HLA-B p.Tyr171His; OR = 1.47; 95% CI, 1.30-1.66; *P* = 1.2 × 10^−9^) (eTable 2 in the Supplement). HLA-B p.Tyr171His is located at peptide binding groove, which would have an important role in immune-related diseases (Figure 3).

According to Allele Frequency Net Database (see eAppendix in the Supplement), HLA-B*52:01 is a common HLA-B allele in the Japanese population (frequency = 0.11). However, this allele has lower frequencies in the Sub-Saharan African population (frequency = 0.016) and European population (frequency = 0.014).

## Discussion

In this study, we conducted the HLA risk fine-mapping analysis by using the Japanese population-specific reference panel.^11^ Our findings suggest that HLA-B*52:01, a common variant of HLA class I genes, may explain the CC risk associated within the MHC region among the Japanese population.

In the previous CC GWAS,^7^ the association of the MHC region with CC was the strongest among the 3 genome-wide significant associations. The SNV-based heritability including the MHC region was estimated as 0.082, and the MHC region alone explained 7.9% of the total heritability.^7^ Because the MHC region here was defined as 24 to 36 Mb on chromosome 6, which accounts for approximately 0.40% of the genome in length, it was suggested that the MHC region has an approximately 21-fold contribution to the development of CC compared with non-MHC region on average.^7^ On the other hand, the detailed analysis regarding which genes within the MHC region explain this association has yet to be fully investigated.

Although several class II HLA alleles have been reported to have associations with CC by candidate gene–based approaches,^13,14,15^ this is, to our knowledge, the first study among the Japanese population that comprehensively assessed the risk of both class I and class II HLA genes. Furthermore, our results suggest that an HLA-B*52:01 allele is associated with CC. Along with its common allele frequency among the Japanese population, the HLA imputation method using the large-scale Japanese population–specific reference panel enabled us to obtain high imputation quality of HLA-B*52:01 allele (estimated *r*^2^ = 0.98). Thus, it is unlikely that the CC risk associated with HLA-B*52:01 was overestimated because of inaccurate imputation.

The HLA-B*52:01 allele has diverse allele frequency spectra around world-wide populations (Figure 2). According to the Allele Frequency Net Database (see eAppendix in the Supplement), this allele is more common in the Japanese population (frequency = 0.11). On the other hand, this allele has lower frequencies in the Sub-Saharan African population (frequency = 0.016) and European population (frequency = 0.014). This suggested that HLA-B*52:01 is responsible for CC in Japanese in a population-specific manner.

We further identified CC risk associated with the HLA-B amino acid variant (p.Tyr171His; Figure 3 and eTable 2 in the Supplement). The previous study among mix populations of Australia, the US, and Europe suggested that the CC risk associated with amino acid variant of HLA-B (Trp156).^21^ The residue Trp156 is located at the peptide-binding groove as well as Tyr171 identified in our study. Because class I HLA molecules are thought to be responsible for presenting endogenous peptides, it is suggested that HLA-B molecules play an important role in presenting HPV-derived peptides in HPV-integrated cells. Immune cells can be differently activated by the presented peptides, leading to failure in clearance of the infected cells. Along with the difference in the observed HPV genotypes among different populations,^23^ these results suggested that specific genotypes of HPVs might have an advantage of escaping from immune responses. Specific viral antigens presented on class I HLA molecules could be different depending on common HLA alleles in each population. Combined assessment of individuals’ HLA alleles and HPV genotypes is warranted to further elucidate the detail in disease biology.

### Limitations

Although the current study is the largest in the Japanese population, one limitation includes the lack of replication study. This is the task for our future study. Also, functional assessment of biological mechanisms at molecular levels should be further investigated.

## Conclusions

In conclusion, our fine-mapping analysis using HLA imputation with the large-scale population-specific reference panel highlighted the association of novel class I HLA risk allele of HLA-B*52:01, which is population-specific. Our study underscores the importance of accumulation of genetic studies from a variety of populations to provide deeper insight into the disease biology of CC.

## Notes

Supplement. **eTable 1.** Associations of the Previously Reported HLA Variants for Uterine Cervical Cancer Risk in Japanese **eTable 2.** Associations of the HLA Amino Acid Polymorphism for Uterine Cervical Cancer Risk in Japanese **eAppendix.** URLs
