# Supplementary material for: Fine Mapping of the Major Histocompatibility Complex Region and Association of the HLA-B*52:01 Allele With Cervical Cancer in Japanese Women
Source: JAMA Netw Open. 2020 Oct 29;3(10):e2023248. doi: 10.1001/jamanetworkopen.2020.23248 (PMC7596586; doi:10.1001/jamanetworkopen.2020.23248)
Supplement: Supplement. — eTable 1. Associations of the Previously Reported HLA Variants for Uterine Cervical Cancer Risk in Japanese eTable 2. Associations of the HLA Amino Acid Polymorphism for Uterine Cervical Cancer Risk in Japanese eAppendix. URLs [file jamanetwopen-e2023248-s001.pdf]

Supplemental Online Content

Masuda T, Ito H, Hirata J, et al. Fine mapping of the major histocompatibility complex region and association of the HLA-B*52:01 allele with cervical cancer in Japanese women. *JAMA Netw Open*. 2020;3(10):e2023248. doi:10.1001/jamanetworkopen.2020.23248

eTable 1. Associations of the Previously Reported HLA Variants for Uterine Cervical Cancer Risk in Japanese
eTable 2. Associations of the HLA Amino Acid Polymorphism for Uterine Cervical Cancer Risk in Japanese
eAppendix. URLs

This supplemental material has been provided by the authors to give readers additional information about their work.

© 2020 Masuda T et al. *JAMA Network Open.*

eTable 1. Associations of the Previously Reported HLA Variants for Uterine Cervical Cancer Risk in Japanese

	Frequency in previous studies		Previous report			Frequency in current study		Current study pre-conditioning		Current study post-conditioning on HLA-B*52:01	
**Variant**	**UCC**	**Control**	**OR (95% CI)**	***P***	**Reference**	**UCC**	**Control**	**OR (95% CI)**	***P***	**OR (95% CI)**	***P***
A*02:06	0.126	0.269	NA	6.0×10^{-3}	Hosono S, et al. *J Epidemiol* 2010	0.074	0.088	0.83 (0.65-1.05)	1.2×10^{-1}	1.22 (0.88-1.43)	3.4×10^{-1}
A*03:01	0.292	0.238	1.4 (1.1-1.9)	2.0×10^{-2}[a]	Madeleine MM, et al. *Cancer Res* 2008	0.003	0.003	0.78 (0.22-2.78)	7.1×10^{-1}	1.20 (0.34-4.27)	7.8×10^{-1}
B*07	NA	0.162	1.32 (NA)	1.9×10^{-9}	Leo PJ, et al. *PLoS Genet* 2017	0.067	0.054	1.22 (0.99-1.52)	6.6×10^{-2}	0.77 (0.62-0.95)	1.6×10^{-2}
B*07:02	NA	0.160	1.31 (NA)	3.9×10^{-9}	Leo PJ, et al. *PLoS Genet* 2017	0.067	0.054	1.22 (0.99-1.52)	6.6×10^{-2}	0.77 (0.62-0.95)	1.6×10^{-2}
B*15	NA	0.072	0.64 (NA)	1.6×10^{-9}	Leo PJ, et al. *PLoS Genet* 2017	0.092	0.113	0.78 (0.64-0.95)	1.2×10^{-2}	1.20 (0.99-1.46)	6.7×10^{-2}
B*15:01	NA	0.065	0.63 (NA)	4.4×10^{-9}	Leo PJ, et al. *PLoS Genet* 2017	0.056	0.078	0.69 (0.54-0.88)	3.1×10^{-3}	1.36 (1.06-1.74)	1.6×10^{-2}
B*15:01	0.063	0.113	0.6 (0.4-0.9)	2.0×10^{-2}[a]	Madeleine MM, et al. *Cancer Res* 2008	0.056	0.078				
B*44:02	0.229	0.139	1.9 (1.4-2.7)	1.0×10^{-4}[a]	Madeleine MM, et al. *Cancer Res* 2008	0.005	0.005	1.00 (0.46-2.16)	1.0×10^{0}	0.95 (0.44-2.05)	8.9×10^{-1}
Cw*05:01	0.204	0.143	1.6 (1.2-2.3)	5.0×10^{-3}[a]	Madeleine MM, et al. *Cancer Res* 2008	0.005	0.005	0.97 (0.45-2.12)	9.4×10^{-1}	0.98 (0.45-2.13)	9.5×10^{-1}
DRB1*07	0.253	0.064	2.31 (1.08-4.95)	4.1×10^{-2}	Hu JM, et al. *Futur Oncol* 2018	0.002	0.003	0.37 (0.09-1.49)	1.6×10^{-1}	2.50 (0.62-10.02)	2.0×10^{-1}
DRB1*09:01	0.177	0.267	0.59 (0.44-0.79)	3.0×10^{-4}	Matsumoto K, et al. *Cancer Sci* 2015	0.156	0.144	1.07 (0.92-1.49)	4.1×10^{-1}	0.89 (0.77-1.04)	1.3×10^{-1}
DRB1*11	0.107	0.038	0.30 (0.15-0.63)	1.0×10^{-3}	Hu Y, et al. *J Cancer* 2017	0.035	0.025	1.43 (1.05-1.94)	2.3×10^{-2}	0.66 (0.48-0.90)	7.8×10^{-3}
DRB1*11:01	0.121	0.061	2.1 (1.3-3.2)	1.0×10^{-3}[a]	Madeleine MM, et al. *Cancer Res* 2008	0.035	0.025	1.43 (1.05-1.94)	2.3×10^{-2}	0.66 (0.48-0.90)	7.8×10^{-3}
DRB1*13	0.007	0.069	10.79 (2.45-46.95)	$<1.0 \times 10^{-3}$	Hu Y, et al. *J Cancer* 2017	0.051	0.078	0.63 (0.49-0.80)	1.6×10^{-4}	1.51 (1.18-1.93)	9.4×10^{-4}
DRB1*13	NA	0.106	0.69 (NA)	1.1×10^{-9}	Leo PJ, et al. *PLoS Genet* 2017	0.051	0.078				
DRB1*13:01	NA	0.055	0.62 (NA)	2.9×10^{-8}	Leo PJ, et al. *PLoS Genet* 2017	0.006	0.006	1.00 (0.51-1.95)	1.0×10^{0}	0.95 (0.48-1.86)	8.8×10^{-1}

© 2020 Masuda T et al. *JAMA Network Open.*

DRB1*13:01		0.024	0.122		0.18 (0.02-0.75)	1.2×10^{-2}	Chambuso R, et al. *J Cancer* 2019		0.006	0.006						
DRB1*13:02		0.057	0.118		0.44 (0.28-0.71)	3.0×10^{-4}	Matsumoto K, et al. *Cancer Sci* 2015		0.044	0.071		0.60 (0.46-0.77)	9.0×10^{-5}		1.59 (1.22-2.06)	5.0×10^{-4}
DRB1*13:02		NA	NA		NA	3.0×10^{-2}[b]	Matsumoto K, et al. *Int J Gynecol Cancer* 2012		0.044	0.071						
DRB1*13:02		0.052	0.091		0.5 (0.3-0.9)	3.0×10^{-2}[a]	Madeleine MM, et al. *Cancer Res* 2008		0.044	0.071						
DRB1*15		NA	0.153		1.42 (NA)	1.4×10^{-11}	Leo PJ, et al. *PLoS Genet* 2017		0.217	0.182		1.32 (1.16-1.50)	3.2×10^{-5}		0.98 (0.82-1.17)	7.9×10^{-1}
DRB1*15:01		NA	0.140		1.43 (NA)	5.6×10^{-12}	Leo PJ, et al. *PLoS Genet* 2017		0.075	0.081		1.01 (0.83-1.24)	9.0×10^{-1}		0.94 (0.77-1.16)	5.6×10^{-1}
DQB1*02		0.123	0.212		1.91 (1.21-3.02)	5.0×10^{-3}	Hu Y, et al. *J Cancer* 2017		0.001	0.004		0.27 (0.07-1.14)	7.4×10^{-2}		3.40 (0.82-1.41)	9.3×10^{-2}
DQB1*02		0.340	0.418		0.7 (0.6-1.0)	4.0×10^{-2}[a]	Madeleine MM, et al. *Cancer Res* 2008		0.001	0.004						
DQB1*03		0.688	0.569		1.67 (1.11-2.50)	1.4×10^{-2}	Hu JM, et al. *Futur Oncol* 2018		0.360	0.370		0.94 (0.84-1.06)	3.0×10^{-1}		0.98 (0.87-1.10)	7.5×10^{-1}
DQB1*03:01		0.169	0.040		4.66 (1.85-11.72)	2.0×10^{-3}	Chambuso R, et al. *J Cancer* 2019		0.105	0.108		0.98 (0.82-1.17)	8.3×10^{-1}		0.96 (0.80-1.15)	6.7×10^{-1}
DQB1*03:01		0.401	0.314		1.5 (1.1-1.9)	3.0×10^{-3}[a]	Madeleine MM, et al. *Cancer Res* 2008		0.105	0.108						
DQB1*03:19		0.013	0.100		0.11 (0.003-0.74)	1.1×10^{-2}	Chambuso R, et al. *J Cancer* 2019		NA	NA		NA	NA		NA	NA
DQB1*06:01		0.188	0.096		0.46 (0.28-0.76)	2.0×10^{-3}	Hu Y, et al. *J Cancer* 2017		0.223	0.178		1.32 (1.16-1.50)	3.2×10^{-5}		0.96 (0.81-1.14)	6.5×10^{-1}
DQB1*06:02		NA	0.141		1.44 (NA)	4.5×10^{-12}	Leo PJ, et al. *PLoS Genet* 2017		0.072	0.078		1.02 (0.83-1.25)	8.7×10^{-1}		NA	NA
DQB1*06:02		0.324	0.149		2.68 (1.45-4.96)	2.0×10^{-3}	Chambuso R, et al. *J Cancer* 2019		0.072	0.078						
DQB1*06:03		NA	0.057		0.63 (NA)	4.2×10^{-8}	Leo PJ, et al. *PLoS Genet* 2017		0.006	0.006		1.00 (0.51-1.97)	9.9×10^{-1}		0.94 (0.48-1.85)	8.6×10^{-1}
DQB1*06:04		0.052	0.116		0.41 (0.26-0.68)	1.0×10^{-4}	Matsumoto K, et al. *Cancer Sci* 2015		0.040	0.065		0.59 (0.45-0.78)	1.8×10^{-4}		1.60 (1.21-2.11)	9.6×10^{-4}

Abbreviations: UCC, uterine cervical cancer; OR, Odds ratio; CI, confidence interval; NA, not available.

Associations reported to be significant in each study are listed. Those satisfied the genome-wide significance threshold is limited. The underlined ORs are probably reciprocal. [a]The Holm P value correcting for multiple comparisons were shown. [b]The P value was determined by log-rank test.

© 2020 Masuda T et al. *JAMA Network Open.*

eTable 2. Associations of the HLA Amino Acid Polymorphism for Uterine Cervical Cancer Risk in Japanese

**Amino acid position**		**Previous report omnibus *P***		**Current study omnibus *P* pre-conditioning**	**Current study omnibus *P* post-conditioning on HLA-B*52:01**
B_156		1.0×10^{-15}		4.0×10^{-4}	0.067
B_171		NA		1.2×10^{-9}	0.012
DRB1_13		5.2×10^{-17}		1.0×10^{-4}	0.078
DRB1_71		1.3×10^{-17}		2.3×10^{-6}	0.0067

Abbreviations: NA, not available. Amino acid polymorphisms previously reported to have significant association with uterine cervical cancer in reference 21 are compared to the current study.

© 2020 Masuda T et al. *JAMA Network Open.*

eAppendix. URLs

1000 Genome Project, http://www.internationalgenome.org/

EIGENSOFT v6.1.4, https://www.hsph.harvard.edu/alkes-price/software/

PLINK 1.9b3.3, https://www.cog-genomics.org/plink2

Minimac3, https://genome.sph.umich.edu/wiki/Minimac

Eagle, https://data.broadinstitute.org/alkesgroup/Eagle/

Allele Frequency Net Database, http://www.allelefrequencies.net/

© 2020 Masuda T et al. *JAMA Network Open.*
